# A Comprehensive Mixture of Tobacco Smoke Components Retards Orthodontic Tooth Movement via the Inhibition of Osteoclastogenesis in a Rat Model

**DOI:** 10.3390/ijms151018610

**Published:** 2014-10-15

**Authors:** Maya Nagaie, Aki Nishiura, Yoshitomo Honda, Shin-Ichi Fujiwara, Naoyuki Matsumoto

**Affiliations:** 1 Department of Orthodontics, Graduate School of Dentistry, Osaka Dental University, 8-1, Kuzuha Hanazonocho, Hirakata, Osaka 573-1121, Japan; E-Mails: mayayam0812@gmail.com (M.N.); naoyuki@cc.osaka-dent.ac.jp (N.M.); 2 Institute of Dental Research, Osaka Dental University, 8-1, Kuzuha Hanazonocho, Hirakata, Osaka 573-1121, Japan; 3 Department of Chemistry, Osaka Dental University, 8-1, Kuzuha Hanazonocho, Hirakata, Osaka 573-1121, Japan; E-Mail: fujiwara@cc.osaka-dent.ac.jp

**Keywords:** tobacco, bone, osteoclast, tooth movement, orthodontics, nicotine

## Abstract

Tobacco smoke is a complex mixture of numerous components. Nevertheless, most experiments have examined the effects of individual chemicals in tobacco smoke. The comprehensive effects of components on tooth movement and bone resorption remain unexplored. Here, we have shown that a comprehensive mixture of tobacco smoke components (TSCs) attenuated bone resorption through osteoclastogenesis inhibition, thereby retarding experimental tooth movement in a rat model. An elastic power chain (PC) inserted between the first and second maxillary molars robustly yielded experimental tooth movement within 10 days. TSC administration effectively retarded tooth movement since day 4. Histological evaluation disclosed that tooth movement induced bone resorption at two sites: in the bone marrow and the peripheral bone near the root. TSC administration significantly reduced the number of tartrate-resistant acid phosphatase (TRAP)-positive osteoclastic cells in the bone marrow cavity of the PC-treated dentition. An *in vitro* study indicated that the inhibitory effects of TSCs on osteoclastogenesis seemed directed more toward preosteoclasts than osteoblasts. These results indicate that the comprehensive mixture of TSCs might be a useful tool for detailed verification of the adverse effects of tobacco smoke, possibly contributing to the development of reliable treatments in various fields associated with bone resorption.

## 1. Introduction

The number of orthodontic patients has been increasing remarkably in the past three decades, reaching approximately 5 million in North America alone [1]. Notably, 25% of these patients are adults [1], suggesting that orthodontic therapy is no longer unusual for this population [2,3], and more attention should be focused on risk factors such as age and psychosocial factors [2]. Nevertheless, compared to other risk factors, the effects of tobacco smoke on tooth movement have been addressed by very few studies. One study reported that nicotine, a major component of tobacco, accelerates tooth movement in an experimental rat model [4]. Conversely, another study reported that nicotine causes adverse effects on tooth movement in histological examinations in the same rat model [5]. These discrepancies clearly indicate that limited information is available on the function of tobacco smoke in orthodontic tooth movement, although further information is imperative for establishing reliable orthodontic treatment.

Orthodontic tooth movement is induced by the prolonged application of mechanical force [6]. Bone and periodontal ligament remodeling are closely associated with this movement process [7]. Bone remodeling is generally controlled by harmonious bone formation and resorption associated with cells such as osteoblasts, osteocytes, and osteoclasts [8]. Osteoclasts are pivotal in bone resorption, while osteoblasts and osteocytes regulate osteoclastogenesis with the molecules macrophage colony-stimulating factor (M-CSF) and receptor activator of nuclear factor kappa-B (NF-κB) ligand (RANKL) [8]. Mechanical force is a crucial stimulus of bone metabolism, whereas it shows a dual function in bone remodeling: adequate stress maintains the bone mass, whereas excess stress induces bone resorption by enhancing osteoclast number and activity [9]. Orthodontic tooth movement substantially uses this bone resorption process, and the degree of tooth movement correlates with osteoclast number at the bone resorption site [10].

Smoking is a deleterious risk factor for systemic and oral diseases [11,12]. Thus, the effects of chemicals in tobacco smoke on bone biology have been widely investigated [4,5,11,13,14,15,16]. However, most studies have investigated a single chemical in smoke, such as nicotine [11,16], benzo(a)pyrene [13,14,15], or 7,12-dimethylbenz(a)anthracene [14]. Although tobacco smoke is a complex mixture containing >5000 chemicals, including nicotine and tar [17], no study has used a comprehensive mixture of tobacco smoke components (TSCs) and evaluated its effect on bone resorption, especially that induced by a mechanical stress highly related to orthodontic tooth movement.

We recently used the original scavenging method to isolate a comprehensive mixture of TSCs containing nicotine and tar [18]. The present study was designed to clarify whether the comprehensive mixture of TSCs hindered mechanical stress-induced bone resorption, thereby retarding experimental tooth movement *in vivo*. To evaluate the detailed mechanism, we examined the effects of TSCs on osteoclastogenesis *in vitro* using a rat osteoblastic cell line UMR106 and primary preosteoclasts.

## 2. Results

**Figure 1:**
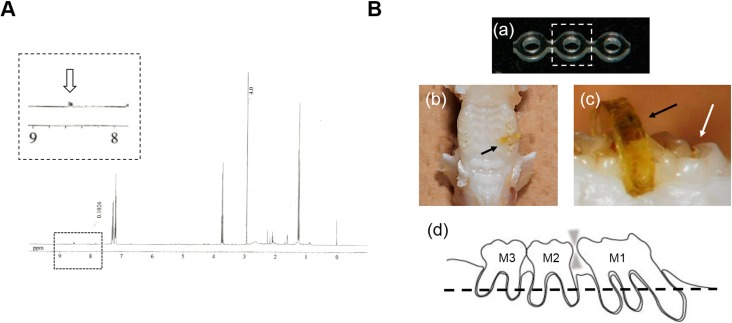
Prepared TSCs and experimental tooth movement model. (**A**) ^1^H NMR spectra of the comprehensive mixture of tobacco smoke components (TSCs) containing tar and nicotine. The arrow in the magnified area represents the specific spectra of nicotine; (**B**) Representative images of the experimental tooth movement model in the rat. (**a**) Intact power chain (PC). Broken-line square: part used in the animal experiments; (**b**,**c**) Macro-images obtained from occlusal and lateral views. Black arrow: PC discolored by TSC administration. White arrow: first molar (M1). PC was inserted unilaterally between the first and second molars; (**d**) Scheme of the sagittal two-dimensional section of PC-inserted dentition in maxillary alveolar bone. The broken line represents a plane used in the following micro-computed tomography (μCT) image and histological section in Figure 4.

Figure 1A shows the ^1^H NMR spectra of isolated TSCs, suggesting the specific peaks for nicotine and tar [18]. TSCs contained approximately 7.9% nicotine (*w*/*w* %). Figure 1B shows representative images of the experimental tooth movement model in rat. To verify TSC uptake in the rats, we confirmed the presence of cotinine, a major metabolic product of nicotine, in urine from the treated rats in a pilot study. Cotinine was detectable from the urine of TSC-treated rats.

**Figure 2:**
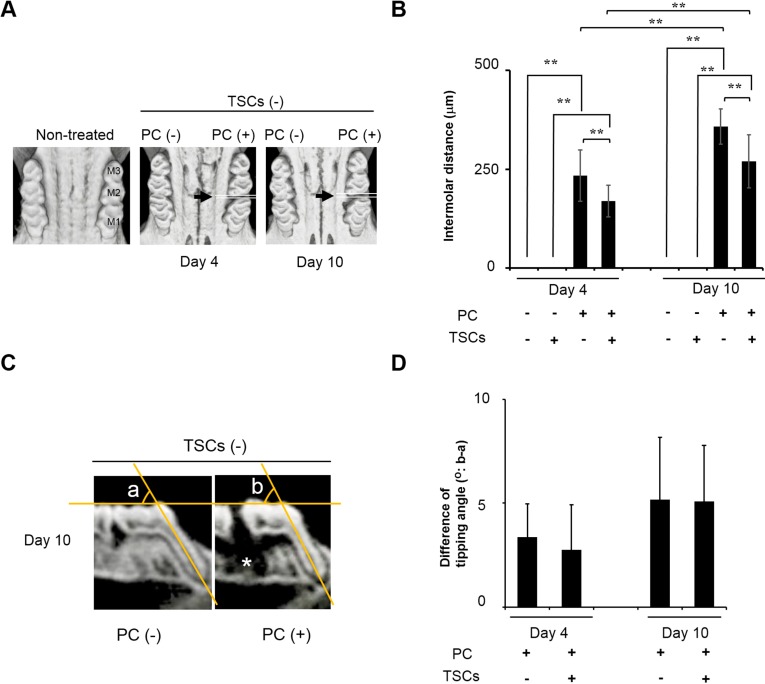
The comprehensive mixture of tobacco smoke components (TSCs) retarded experimental tooth movement. M1: first molar. M2: second molar. M3: third molar. PC: power chain. (**A**,**C**) μCT images of the maxillary bone observed from occlusal and lateral views; (**A**) Black arrows represent the PC insertion site. PC time-dependently separated the molars; (**B**) Quantitative data on intermolar distances between M1 and M2. All data show mean ± s.d. (*n* = 10). ** *p* < 0.01 (ANOVA with Tukey–Kramer test). TSCs significantly retarded experimental tooth movement induced by PC insertion; (**C**) Representative sagittal two-dimensional section of dentition with/without PC insertion. PC insertion caused slight tipping for M1. Angles a and b: representative tipping angles with/without tooth movement used in (**D**); Contralateral side was used as angle a. White asterisk: disappeared alveolar bone adjacent to the distal root of M1; (**D**) The quantitative difference in tipping angles of M1 with/without PC insertion. Data show angle b minus angle a. All data show mean ± s.d. (*n* = 10). There was no significant difference in tipping angles between rats treated with/without TSCs (Student’s *t*-test).

The effect of TSCs on tooth movement is shown in Figure 2. Figure 2A,C represent μCT images of the maximal bone obtained in occlusal and lateral views. PC inserted unilaterally between M1 and M2 effectively induced tooth movement time-dependently, resulting in intermolar gaps within 10 days (Figure 2A). TSC administration significantly reduced the gap distances (Figure 2B). A lateral view suggested that tooth movement was, in part, due to the slant of M1 (Figure 2C). However, there was a negligible difference in the tipping angle in the presence or absence of TSCs, suggesting that tooth movement might be due to bodily tooth movement (Figure 2C,D). The alveolar bone adjacent to the distal root of M1 tended to disappear after PC insertion (Figure 2C, white asterisk).

**Figure 3:**
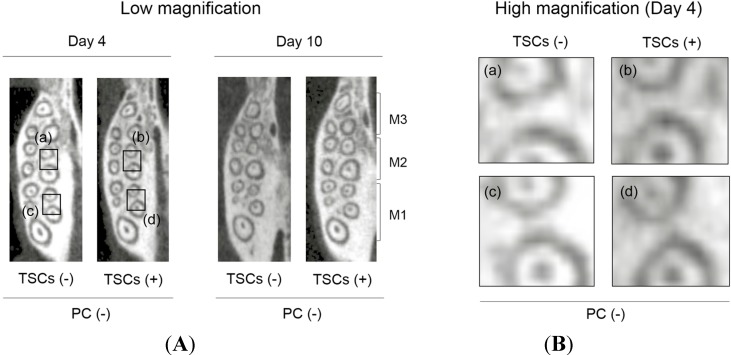
μCT image of alveolar bone without power chain (PC) treatment. (**A**) Low magnification. Squares (**a**–**d**): magnified area; (**B**) High magnification. TSCs: tobacco smoke components. There is negligible difference in radiopacity among the non-PC treated dentitions with/without TSCs. M1: first molar. M2: second molar. M3: third molar.

**Figure 4:**
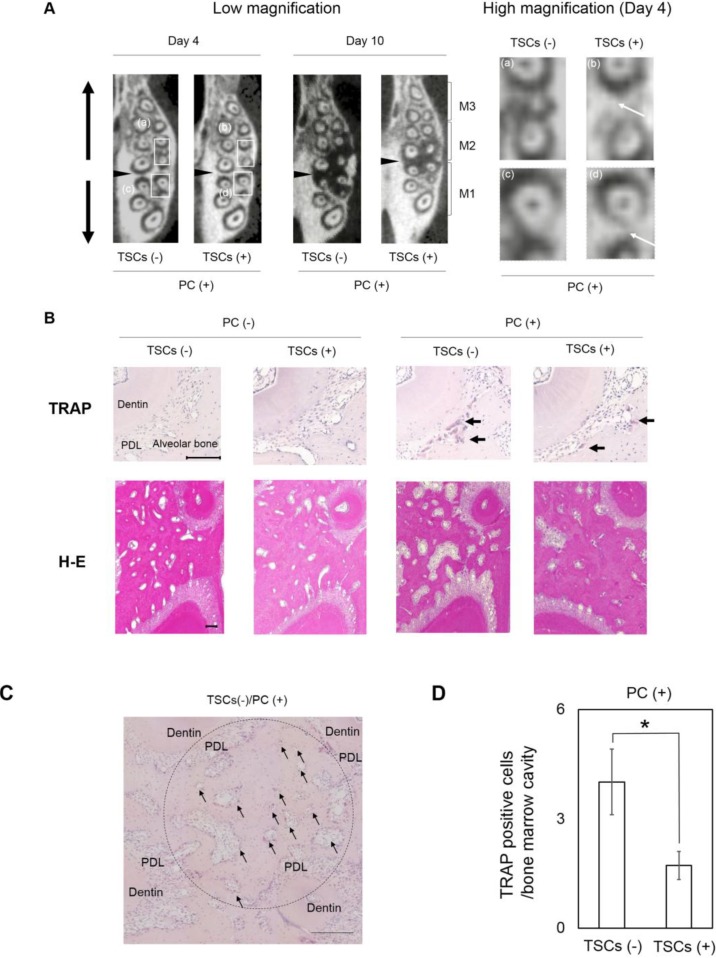
The effects of the tobacco smoke components (TSCs) on bone structure. PC: power chain. (**A**) μCT image of maxillary alveolar bone obtained from axial view. Black arrowheads: PC insertion site. M1: first molar. M2: second molar. M3: third molar. TSC administration impaired the decreased radiopacity induced by PC insertion (white arrows at high magnification). Square (**a**–**d**): magnified area; (**B**) Representative histological sections stained with TRAP and H&E. Scale Bar: 100 μm. TRAP-stained sections represent areas adjacent to the distal root of M1. Black arrows: TRAP-positive cells. H&E-stained sections represent areas of the interradicular septum under M1. PDL: periodontal ligament; (**C**) Representative TRAP-staining of interradicular septum under M1. Round area (ϕ 900 µm) was used to quantify TRAP positive cells in bone marrow cavity two-dimensionally isolated from PDL. Scale Bar: 200 µm. Black arrows: representative bone marrow cavities used for the quantification; (**D**) Quantitative data for the number of TRAP-positive cells in each bone marrow cavity. Data show mean ± s.d. * *p* < 0.05 (Student’s *t*-test). Overall, 51 and 76 cavities for TSCs (−) and TSCs (+) were evaluated for each group.

To verify the mechanism whereby TSC administration hindered tooth movement induced by PC insertion, we evaluated the middle of the maximal alveolar bone by using μCT images in axial view (Figure 3 and Figure 4A). On the basis of the radiopacity reflecting the bone adjacent to the distal and mesial roots of M1 and M2, TSC administration caused negligible differences in the dentitions without PC insertion (Figure 3). Conversely, PC insertion into the non-TSC-treated rats markedly decreased the radiopacity (Figure 4A). TSC administration apparently impaired this reduction, indicating that TSC administration effectively inhibited bone resorption. On histological evaluation using tartrate-resistant acid phosphatase (TRAP) staining, PC insertion recruited TRAP-positive osteoclastic cells for the TSC- and non-TSC-treated rats. However, there were fewer TRAP-positive cells in TSC-treated rats (Figure 4B). Hematoxylin and eosin (H&E) staining revealed that PC insertion increased the size of the bone marrow cavity in the non-TSC-treated rats, while the size was maintained with TSC administration. There were relatively fewer TRAP-positive cells in the bone marrow cavity of the TSC-treated rats (Figure 4C,D). These data suggest that the retardation of tooth movement induced by TSC administration was, in part, due to the impairment of osteoclastogenesis.

**Figure 5:**
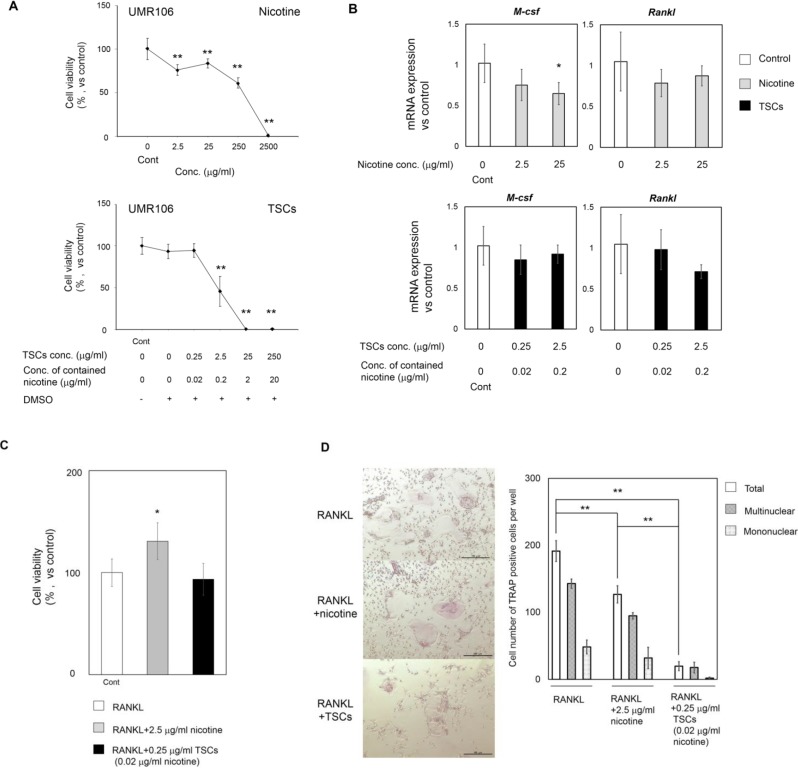
The effects of nicotine and TSC administration against osteoclastogenesis *in vitro*. TSCs: tobacco smoke components. (**A**) Cytotoxicity of nicotine and TSCs to UMR106. Cells were seeded at 6.3 × 10^3^/cm^2^ and subjected to nicotine and TSCs at different concentrations for 9 and 6 days, respectively. All cell numbers were normalized against those treated with control medium; (**B**) *M-csf* and *Rankl* mRNA expression in UMR106. Cells were seeded at 2.8 × 10^4^/cm^2^ and subjected to nicotine and TSCs at different concentrations for 48 h; (**C**) Cytotoxicity of nicotine and TSCs to the primary preosteoclasts. Cells were seeded at 1.9 × 10^5^/cm^2^ and subjected to the adjunct culture medium containing RANKL (15 ng/mL) with/without TSCs and nicotine for 10 days. All cell numbers were normalized against those treated with adjunct culture medium; (**D**) TRAP staining and its quantitative data. Cells were treated as in (**C**). Scale Bar: 100 µm. The total number contains mononuclear and multinuclear TRAP-positive cells. All data show mean ± s.d. (*n* = 3). (**A**–**C**) ** *p* < 0.01, * *p* < 0.05 compared with control (ANOVA with Dunnett’s test) and (**D**) ** *p* < 0.01 (ANOVA with a Tukey–Kramer test).

Periodontal ligament (PDL) cells have been widely investigated in the tooth movement model because of their location and contribution to the induction of osteoclastogenesis [19]. However, our histological data suggested that bone resorption induced by PC occurred strongly not only at the peripheral bone adjacent to PDL but also at the bone marrow (Figure 4B–D). The impairment of osteoclastogenesis in bone marrow seemed to correlate with the retardation of tooth movement. To identify the pivotal cells associated with inferior bone resorption in the bone marrow, we examined the effects of TSCs on osteoclastogenesis in UMR106 and preosteoclasts *in vitro* (Figure 5A,B for UMR106; Figure 5C,D for preosteoclasts). Nicotine was used as a positive control. Figure 5A shows the cytotoxicity of nicotine and TSCs. Nicotine (2.5 mg/mL) and TSCs (25 μg/mL) completely impaired cell viability. TSC administration was almost 100-fold more severely cytotoxic relative to nicotine administration alone. Nevertheless, TSCs had negligible effects on *M-csf* and *Rankl* mRNA expression (Figure 5B). Using the less cytotoxic concentrations of TSCs and nicotine for UMR106 and primary preosteoclasts (Figure 5A,C), we evaluated whether the two substances impaired osteoclastogenesis from the primary preosteoclasts (Figure 5D). TSC administration significantly decreased the number of TRAP-positive cells. Notably, the ratio of multinuclear TRAP-positive cells against total cells increased in the TSC-administered cells. Nicotine concentration contained in the medium with TSCs was approximately 1% of medium with nicotine alone. Some components in TSCs other than nicotine would potently inhibit osteoclastogenesis.

## 3. Discussion

Tobacco is a major risk factor for oral and systemic diseases [11,12]. Nevertheless, the effects of TSC administration on orthodontic tooth movement are not completely known. We demonstrated that TSC administration significantly impaired tooth movement in a rat model, in part, because of the inhibition of osteoclastogenesis.

The history of the tooth movement model spans >100 years [20]. Numerous tools can be applied in the experimental tooth movement model [20]. Compared to other methods, PC may constructively induce an inflammatory response in the periodontal ligament. However, we selected PC to facilitate tooth movement because of its usability and higher stability than other elastic bands and chains, which may be frequently eliminated from the dentition. Meanwhile, it was difficult to quantify the applied mechanical force in the experiment. Given the severe bone resorption presented as low radiopacity in μCT image on day 10 (Figure 4A), the mechanical force used in this study should be higher than the optimal force. In the clinic, orthodontic tooth movement occasionally applies slightly stronger mechanical force than optimal force depending on the type of tooth movement and the type of tooth to be moved. Our data may indicate that tobacco smoke had the same capability to retard tooth movement, even at that higher mechanical force used clinically.

μCT images and histological sections were obtained at a distance from the gingiva to eliminate the intervention of periodontitis (Figure 1B(d) and Figure 4), which may lead to misinterpretation of the results, because it is considered that lipopolysaccharide (LPS), a component of gram-negative bacteria in dental plaque, is a potent stimulant for severe osteoclastogenesis followed by bone loss [19]. Furthermore, Tanaka *et al.* have reported that LPS and nicotine synergistically enhance osteoclastogenesis in mouse cells [11]. However, the comprehensive relationship among periodontitis, tobacco smoking, and orthodontic tooth movement is clinically yet to be explored. The feasibility of orthodontic treatment is still controversial for adult patients with destructive periodontal diseases [21]. Thus, we firstly attempted to evaluate the effects of TSCs on mechanical force-induced bone resorption at the middle of the alveolar bone.

To date, various studies have evaluated the effects of TSCs on bone biology using a single molecule, such as nicotine. In contrast, we utilized the comprehensive mixture of TSCs containing nicotine and tar isolated from our original technique. Our results showed opposite effects on tooth movement compared with those in a previous study using nicotine alone, even in a similar rat model [4]. Benzo(a)pyrene contained in tar interacts with the aryl hydrocarbon receptor, resulting in the induction of osteoclastogenesis in a mouse model [13]. Vornov *et al.* reported that benzo(a)pyrene inhibits osteoclastogenesis in mouse cells *in vitro* by attenuation of RANKL-induced NF-κB nuclear translocation and activation [14,15]. Here, TSCs containing only 0.02 μg/mL nicotine had less effect on the induction of *Rankl* and *M-csf* expression for UMR106, while it significantly inhibited osteoclastogenesis (Figure 5). These results indicate that chemicals in TSCs other than nicotine and benzo(a)pyrene may have a key role in modulating tooth movement through the inhibition of RANKL-induced osteoclastogenesis.

Smoking markedly affects calcium and vitamin D adsorption associated with bone metabolism, and long-term smoking induces inevitable bone loss [22]. Furthermore, bone quality is altered in osteoporosis [23], which may affect the velocity of tooth movement. Given the other functions of tobacco, further examination, especially in view of smoking-induced osteoporosis, would be imperative for identifying the complete mechanisms governing the retardation of tooth movement.

## 4. Experimental Section

### 4.1. TSC Preparation

TSCs were prepared by the scavenging method previously reported, with the original apparatus and technique [18]. TSCs were isolated from 20 cigarettes (Seven Stars, Japan Tobacco, Tokyo, Japan) that contained nicotine (0.6–1.2 mg) and tar (7–14 mg). To confirm the presence of nicotine and tar, we determined ^1^H NMR spectra for the obtained solution using a JNM-400 NMR spectrometer at 400 MHz (JEOL, Akishima, Japan).

### 4.2. Experimental Tooth Movement Model and Uptake of TSCs

All animal experiments strictly followed guidelines approved by the local ethics committee of Osaka Dental University (Approval #13-02040, 29 March 2013). Male, 13-week-old Wistar rats (10 rats per group) were used for the experimental tooth movement model. Briefly, an elastic power chain (PC; Clear Power Chain II, Ormco, Orange, CA, USA) was unilaterally inserted between the first and second maxillary molars (M1 and M2) to induce tooth movement up to day 10 (Figure 1B). The contralateral side was used as a control. Rats were orally administered with basal pure water (control) or 13 µg/mL TSC water containing 1 µg/mL nicotine. The concentration of TSCs was defined according to the following criteria: (1) the concentration of contained nicotine was below the lethal dose (LD_50_) as mentioned in the safety data sheet (50 mg/kg body weight; KantoKagaku, Tokyo, Japan and Thermo Fisher Scientific, Waltham, MA, USA); and (2) the concentration did not induce diarrhea in the rats in a pilot study. Each rat regularly drank 20.1 mL/day on average with a conventional drinking bottle. Water was supplied during the whole experimental period (4 or 10 days). The presence of cotinine in the urine was determined by using a salivary cotinine quantitative enzyme immunoassay kit (Salimetric, State College, PA, USA) and confirmed at days 4 and 10 in the pilot study.

To estimate the amount of tooth movement, we determined the narrowest intermolar distance between M1 and M2 to estimate the amount of tooth movement using μCT images in occlusal view. The angle between the mesial root of M1 and the occlusal plane was considered as a tipping angle. Despite some limitations due to low resolution, we adopted μCT because of the following three reasons: (1) data are preserved even after preparing the histological sections; (2) the adequate angle and position are defined in three-dimensional (3D) images; and (3) good results have been obtained in a previous study [24].

### 4.3. Analysis of Bone Microstructure

The maxillae were analyzed by μCT scanning (SMX-130CT, Shimadzu, Kyoto, Japan). The bone specimens were scanned continuously in 20-μm increments and yielded 512 slices, with a 45-kV tube voltage and a 73-μA tube current. The voxel size was 20 × 20 × 20 μm^3^. After scanning, the image data were transferred to a workstation and were reconstructed in a 3D image analysis system (TRI/3D-Bon, Ratoc System Engineering, Tokyo, Japan).

### 4.4. Histological Evaluation

After μCT analysis, samples were decalcified with 10% EDTA for 2–3 weeks. The decalcified samples were embedded in paraffin and dissected at 3-μm thickness. The sections were stained with H&E and TRAP. Round area (ϕ 900 µm) at the interradicular septum under M1 was used to count “the number of TRAP positive cells in each bone marrow cavity” (five slides for each group). Representative bone marrow cavity images used for the quantification are indicated by black arrows in Figure 4C. To exclude the effect of PDL cells on osteoclastogenesis, bone marrow cavities directly connected to the PDL were excluded from the analysis.

### 4.5. Cell Culture

A rat osteoblastic cell line, UMR106 (CRL-1661), was purchased from ATCC (Manassas, VA, USA) because of its capability to express *Rankl* [25]. The cells were maintained in Dulbecco’s modified Eagle’s medium with 10% fetal bovine serum and antibiotics in a humidified atmosphere of 5% CO_2_ at 37 °C. The cells were treated with conditioned medium containing TSCs and nicotine up to the prescribed date (from 48 h to day 9) for each assay.

### 4.6. Quantitative Polymerase Chain-Reaction Analysis

mRNA levels were determined for *Rankl*, *M-csf*, and *18s*. UMR106 was harvested at 2.8 × 10^4^/cm^2^ and treated with conditioned medium containing 10 nM 1,25(OH)_2_D_3_ (EMD Chemicals Inc., Gibbstown, NJ, USA) with or without TSCs and nicotine for 48 h; total RNA was extracted using an RNeasy Mini Kit (Qiagen Inc., Valencia, CA, USA). Quantitative polymerase chain reaction (PCR) was performed with a Universal ProbeLibrary set and a FastStart Universal Probe Master (Roche Diagnostics, Mannheim, Germany). Expression of *18s* was evaluated using Gene Expression Assays (#4310893E, ThermoFisher Scientific Inc., Waltham, MA, USA). The primers and probes are listed in Table 1. PCR condition was as follows: 10 min at 95 °C, followed by 45 cycles of 15 s at 95 °C and 60 s at 60 °C. A comparative *C*_t_ method was used to calculate mRNA expression levels.

**Table 1:** Primers and probes for qPCR analysis.

mRNA	Sequence		Probe#	Accession#
*Rankl*	Forward	5'-AGACACAGAAGCACTACCTGACTC-3'	2	NM_057149
	Reverse	5'-GGCCCCACAATGTGTTGTA-3'		
*M-csf*	Forward	5'-CAAGGACTATAAGGAACAGAACGAG-3'	55	NM_023981
	Reverse	5'-GAAATTCTTGATTTTCTCCAGCA-3'		

### 4.7. The Effects of TSCs and Nicotine on Osteoclastogenesis

A commercially available osteoclast culture kit (#OSC11, Cosmo Bio, Tokyo, Japan) was used for evaluating the effects of TSCs and nicotine on osteoclastogenesis. Primary preosteoclasts seeded at 1.9 × 10^5^/cm^2^ were subjected to an adjunct culture medium containing 15 ng/mL RANKL with/without TSCs and nicotine up to day 10. TRAP staining was performed according to the instructions.

### 4.8. Cytotoxicity of TSCs and Nicotine

To evaluate the cytotoxicity of TSCs and nicotine, we evaluated cell numbers using a Cell counting kit-8 (Dojindo Laboratories, Kumamoto, Japan). Cells were seeded at 6.3 × 10^3^/cm^2^ for UMR106 and 1.9 × 10^5^/cm^2^ for preosteoclasts.

### 4.9. Statistical Analysis

Data were calculated with the Microsoft excel software statistics package. Statistical significance was assessed by one-way analysis of variance (ANOVA), with Dunnett’s test and Tukey–Kramer test and by Student’s *t*-test.

## 5. Conclusions

Our results indicate that TSC administration significantly retarded experimental tooth movement in a rat model, in part, because of the capability of TSCs to inhibit osteoclastogenesis *in vivo*. Additionally, our *in vitro* study indicated that the inhibitory effect seems to be directed more toward preosteoclasts than osteoblasts. While there have been studies using single molecules in tobacco smoke, we revealed that TSCs containing small amounts of nicotine have a crucial effect on the inhibition. These results would contribute to a reliable treatment in orthodontics therapy and bone diseases. Furthermore, given the inhibitory mechanism of TSCs to osteoclastogenesis, the comprehensive mixture of TSCs is likely to be a useful tool for verifying the synergistic effect of tobacco smoke components in tooth movement and bone biology.
